# The wiring diagram of an entire animal

**DOI:** 10.7554/eLife.108573

**Published:** 2025-08-27

**Authors:** Alex M Winsor, Paul S Katz

**Affiliations:** 1 Organismic and Evolutionary Biology Graduate Program, University of Massachusetts Amherst Amherst United States; 2 Department of Biology and the Neuroscience and Behavior Graduate Program, University of Massachusetts Amherst Amherst United States

**Keywords:** connectome, marine larva, synapse, cell type, volume EM, evolution of the nervous system, *P. dumerilii*

## Abstract

A digital atlas of every cell in a developing marine worm reveals how networks across the body coordinate sensing and movement, and provides insights into the evolution of the nervous system.

**Related research article** Verasztó C, Jasek S, Gühmann M, Bezares-Calderón LA, Williams EA, Shahidi R, Jékely G. 2025. Whole-body connectome of a segmented annelid larva. *eLife* **13**:RP97964. doi: 10.7554/eLife.97964.

Just as a genome is all the genetic information of an organism, a connectome is a map of all the neuronal connections in the brain of an organism (Swanson and Lichtman, 2016). The human brain contains an estimated 10^11^ neurons, with multiple connections between them, so assembling the connectome of the human brain is beyond the scope of our current computational capacity. However, connectomes have been assembled for much smaller animals, such as the fruit fly, *Drosophila melanogaster* (Schlegel et al., 2024).

The gold standard technique for mapping connections between neurons is to preserve the tissue containing the neurons, and then cut this tissue into slices about 1000 times thinner than the width of a human hair. The next step is to use an electron microscope to generate images of the slices. Finally, these images are stacked on top of each other and the objects within them (such as neurons) are reconstructed in three dimensions. Recently this approach was used to reconstruct the connections between the neurons in a sample of just one cubic millimeter of human brain tissue (Shapson-Coe et al., 2024). While this was an impressive feat, a cubic millimeter is less than one millionth of the total volume of the brain, so it is not nearly enough to provide global insights into the human nervous system.

To truly understand neuronal connectivity and how it leads to behavior, one needs to construct a whole-body connectome that includes both the brain and the rest of the nervous system. To date this has only been achieved for two animals, both of which are even smaller than the fruit fly: the roundworm, *Caenorhabditis elegans*, which contains about 1000 cells in its whole body, 302 of which are neurons, (Cook et al., 2019), and the larval tadpole of the sea squirt, *Ciona intestinalis*, which contains only 301 cells, including 177 neurons (Ryan et al., 2016).

Now, in eLife, Gáspár Jékely and colleagues – including Csaba Verasztó and Sanja Jasek as joint first authors – report that they have assembled a connectome for a 72-hour-old larva of the marine worm, *Platynereis dumerilii* (Verasztó et al., 2025). This larva contains around 9000 cells, including 966 neurons. Moreover, unlike *C. elegans* and *C. intestinalis*, it has a segmented body plan (Helm et al., 2018). Furthermore, *P. dumerilii* is an annelid, which places it in the Spiralia clade, whereas *C. elegans* is a nematode, which is in the Ecdysozoa with insects, and *C. intestinalis* is a tunicate, making it a Deuterostome, like us (Figure 1).

**Figure 1.**
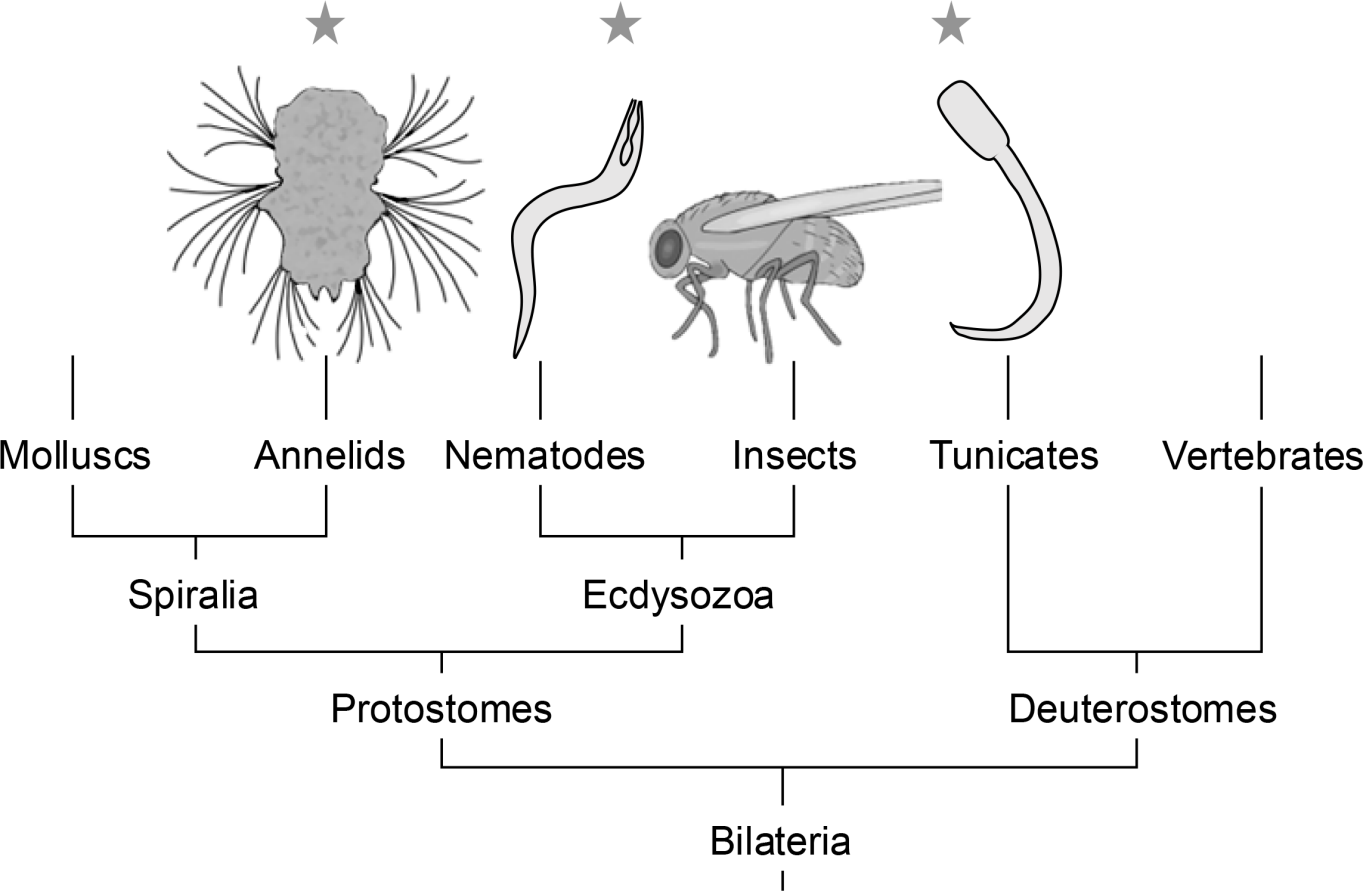
A phylogenetic tree for bilaterally symmetric animals. Whole-body connectomes have been constructed for just three species (indicated by stars): the annelid *P. dumerilii*, the nematode *C elegans*, and the tunicate *C. intestinalis*. A whole-brain connectome has also been assembled for the insect *D. melanogaster*. Between them, these species span three major clades: Spiralia, Ecdysozoa and Deuterostomes. Only partial connectomes are available for molluscs and vertebrates.

The release of the whole-body connectome for *P. dumerilii* is the culmination of over a decade of work by the Jékely group on this microscopic organism, which is only about 200 micrometers in length. By mapping cells, connectivity and gene expression (using various transcriptomic techniques), and by exploiting a range of other molecular tools, these researchers have been able to determine the cellular basis for several behaviors including visual navigation (Randel et al., 2014), ciliary motor coordination (Verasztó et al., 2017), and startle responses (Bezares-Calderón et al., 2018).

What, one might ask, is the value of having a whole-body connectome for an annelid when, unlike *C. elegans*, and *D. melanogaster*, there is not a large community of researchers working on the nervous system of this species? The answer to this question is that there are two ways to approach biological questions: go deep or go broad. The large numbers of researchers working on a small number of ‘model organisms’ are able to go deep into the mechanisms underlying neural development and the production of behavior. However, by going broad and comparing distantly related species, it may be possible to extract the general principles that underlie how all nervous systems function. For example, segmented body plans evolved independently in chordates, which includes vertebrates, arthropods (such as insects), and annelids. Therefore, comparing how the nervous system is organized across body segments in these different examples will help researchers to identify features that are fundamental to segmental control versus features that are specific to just one phylum (Figure 1).

One such general principle is evolution by duplication and divergence. Repeated cell types and linkages are present across the different segments in *P. dumerilii*, suggesting that neuronal circuits were duplicated and repurposed. Investigations of hox genes, which control the body plan of embryos, show that many segments are duplicated during development (He et al., 2018). This phenomenon – which is called segmental homology – has been studied for many years in crustaceans and other segmental animals (Mittenthal and Wine, 1978), and its genetic basis is well studied in insects (Doe and Scott, 1988), and in another annelid, the leech (Weisblat and Kuo, 2014). Having a complete connectome allows a further dissection into how segments are wired with other segments, and how they are specialized along the animal.

The whole-body connectome also provides evidence for understanding evolutionary origins of modern forms. A nineteenth century model posited that the embryonic blastopore is divided into mouth and anus through fusion of the lateral blastopore lips (Nielsen, 2019). The nerve cords then evolved from extensions of the nerve ring around the mouth. The connectome for *P. dumerilii* supports this model.

Combining the new connectome with transcriptomic data on gene expression has allowed Verasztó et al. to go deep into the organization of function in the nervous system of *P. dumerilii*. They find that its brain is organized into distinct zones calls neuropils, where there are many connections between neurons. The cell bodies, which are sites of cell metabolism and genetic storage, form a rind that encapsulates the neuropils. Clusters of sensory cells, such as those that respond to touch and light, flank the brain. Mushroom bodies – structures that enable odor learning in other species – are found deeper inside the brain. Numerous sensory cells project to these structures, compressing the incoming information, before expanding and distributing it elsewhere.

Beyond the brain, connectivity continues along a ventral bundle of nerves that traverses the head to the body segments, forming a nerve cord. Along this tract, another pathway called the mechanosensory girdle receives input direct from mechano-sensors, and projects to secretory cells. Other cells span the body, suggesting an unexplored link between mechanosensation, movement, and internal regulation.

Leveraging a network analysis developed by Google for ranking webpages, based on the number of other webpages linking to them, Verasztó et al. – who are based at the Universities of Exeter, Heidelberg and Bristol, and the EPFL in Lausanne – identified important clusters, or nodes, of connected cells. These nodes formed a highly structured arrangement: sensory cells relayed information to interneuron messengers, which then connected to ‘effector’ cells responsible for carrying out actions, such as movement across distant body parts. There was also significant recurrent connectivity.

Neuroscience is being transformed by connectomics and cellular transcriptomics. For example, the nascent field of comparative connectomics is addressing how evolutionary changes in wiring affect behavior (Cook et al., 2025; Roberts et al., 2025). Having access to whole-body connectomes for more species will enhance our understanding of the general principles that govern how nervous systems function, and provide new insights into the evolution of neural circuits and behavior.
